# Chemistry and personalized medicine – the research and development future of Europe

**DOI:** 10.3325/cmj.2012.53.291

**Published:** 2012-08

**Authors:** Gunars Duburs, Denis Neibecker, Neven Žarković

**Affiliations:** 1 Latvian Institute of Organic Synthesis, Laboratory of Membrane Active Compounds, Riga, Latvia gduburs@osi.lv; 2 CNRS Laboratoire de Chimie de Coordination, Toulouse, France; 3 Laboratory for Oxidative Stress, Ruđer Bošković Institute, Zagreb, Croatia

## Personalized medicine and policy to be implemented: the European chemist point of view

Personalized medicine may represent a dramatic change of paradigm in the medium-term future. For a chemist, personalized medicine means the definition and understanding of any disease on molecular level for each individual or group of individuals (personalized diagnosis) ideally leading to the design of a drug that efficiently counteracts or prevents any molecular dysfunction, ie, a personalized drug without side effects.

The interdisciplinary research required for personalized medicine should overcome a myriad of obstacles not the least being to find specific biomarkers and targets for each individual or group of individuals suffering from a given disease. Chemists enter then into action and will model/design drugs and drug delivery pathways for a personalized therapy. They will either tap into the numerous drugs candidates, which were abandoned at some stage of clinical trials, or synthesize new drugs, mainly those “small molecules” mimicking the activity of natural products.

This view has obvious economic, ethical, and social implications, beyond scientific challenges. All stakeholders will have to take them into account. Policy makers will have to examine all disciplines of regulatory science among which the thorny economics (cost-benefit analysis of specific research and development projects) are of paramount importance and critical to the development of personalized medicine.

Thus competing/conflicting interests of “drugs producers” and “drugs payers” need to be reconciled. It requires resolving intricate issues, which include how to advance translational medical science, how to “reward” new findings on old drugs, and how to deal with intellectual property rights when new products and approaches are incorporated into clinical practice as the results of an interdisciplinary endeavor, very often not stemming from a single country. As a corollary, there is as well European Patent question, which looks a bit of a holy grail, and ultimately the most important one: is it cost-effective for the very different health care systems to uptake personalized medicine?

From the chemist’s point of view, these are several of the key policy issues needing to be addressed and answered to help personalized medicine have a bright future, that is for physician/clinician to prescribe the right drug at the right dose at the right time for every person.

## Medicinal chemistry and personalized medicine as they are and as they might be

Medicinal chemistry comprises several scientific disciplines: organic chemistry, bioorganic chemistry, physical organic chemistry, biochemistry, pharmacology, toxicology, molecular biology, analytical chemistry, engineering, genetics, etc. Nowadays, this complex approach is significantly developing and allows gaining a novel level – personalized medicine. It means: choice of a drug and its use regime should fit every individual specifically, so efficacy of medicinal treatment would improve significantly. Such a progress is possible thanks to a novel approach in many branches, eg, nowadays polypharmacology is developing fast. It means that pleiotropic properties of compounds, which were treated negatively for a long time, considered in many cases as side effects, are reconsidered gradually as desirable bioactivities. Therefore, exclusively selective compounds that have been a quest for decades are not any more considered as golden standards. On the contrary, many well known, powerful, and successful drugs turned out to be “dirty.” They are beneficial since due to their pleiotropic properties they regulate several signaling pathways, which was not realized when they were introduced as drugs with a single activity principle.

The European Cooperation in Science and Technology (COST) conference “Personalised Medicine: Better Healthcare for the Future – A Rational Approach Focusing on Bioinformatics, Medicinal Chemistry and Medicine” was a significant event to establish new principles for the medicine of the future (1). It is a milestone in the fast development of medicinal chemistry and drug development. This strategic COST conference fits the general mission of the COST goals very well: to pick up novel emerging features and principles in science, to support them, and to bring attention of society on the most important novelties. The conference demonstrated the importance of collaboration of many specialists to create innovative products for health care, and food, vitamins, nutritional supplements, as well as high tech materials (2). A series of 18 interviews made on COST Strategic Conference deliver the take-home message from different stakeholders (policy makers, patient associations, scientists, and clinicians) (*http://www.youtube.com/playlist?list=PL08CF13F5A0482A2B&feature=plcp*). Prof. Alain van Gool (coordinator Personal medicine, Netherlands Organization for Applied Scientific Research) turned attention to the relevance of system biology: to monitor the effect of interventions on the human system as a whole and timely adjust when needed (3). When organism turns from healthy state to disease (eg, diabetes, its complications, including nephropathy, brain disorders, cardiovascular events, etc), gradual disturbances of multiple equilibriums occur. Therefore, several specialists should participate in studies of medicinal chemistry and personalized medicine. The potential for multi-target drugs is significant; it coincides with evolvement of novel drug discovery paradigm of designed polypharmacology. Such innovative task will need to involve coordinated collaboration of many specialists. Optimizing preconditions for that is “a must” both for scientists and for policy makers, who should create jointly functional and trusty networking links between academia and industry, both on regional and on global level supporting research and development (4).

## Personalized bio-interdisciplinarity

The past decade in science may be considered as a period of adjustment to the dynamic processes of globalization associated with overall crises; moral, political, and financial. Similar processes happened several times in the 20th century, but the question of optimal strategic approach to solve the crisis has never before been science policy-dependent as nowadays. Therefore, again, as in the seventies, the time is right for science to “functionalize policy” by providing and influencing policy makers and decision makers. The COST, being born in the period of global turbulences forty years ago, might be the optimal system to fulfill such a demanding goal. As it was successful in overcoming the “iron curtain,” COST could be successful in providing interdisciplinarity in science for a better, healthy life, which is of highest importance for the modern, aging society.

It is important to underline the fact that chemistry is a major industry in Europe, which is a global leader in chemistry and related technologies. However, global markets and globalized sciences do not make optimal preconditions to develop further bio-interdisciplinarity for the healthy life in Europe. Although globalization implies integration processes, European diversities still represent obstacles for the set up of the European Research Area, while global economies do not support defining pan-European research and development strategy. The vision of Europe 2020, with a flagship initiative of Innovation Union, does not appear realistic without the fast and affordable European patenting system that could allow the common principle of “first file then publish” to be daily practice for researchers in Europe. Europe has to be an example of wisely using interdisciplinarity in science for the better world of stable research and development and healthy life.

The strategic COST conference in Larnaca clearly pointed to the need of bringing policy makers together with the prominent scientists oriented to the interdisciplinarity. Modern concepts of integrative medicine are based on interdisciplinarity of life sciences. In the center of this stands a healthy modern man, considered as a valuable member of society giving optimal personal contribution to all. However, the average age of modern European is above 40, now with expected life expectancy around 80. Thus, the average healthy Europeans in 2012 already develop chronic stress- and age-associated disorders. In 2030-2035, they will be chronically ill and will stay so for 15-20 years at least. To make the perspective even darker we have to admit that we still do not know how to prevent the major chronic diseases of the modern man, how to diagnose them in time, and how to treat them optimally.

As many times before, chemistry and molecular technologies are at the front of changes combining green chemistry and mimetic chemistry with medicinal chemistry and nanotechnologies. The respective COST Domain is thus flooded with emerging interdisciplinarity in many Actions like in epigenetics and theragnostics, linking them with major tasks of modern biomedicine such as cancer stem cells and neurodegenerative disorders. Supporting such interdisciplinarity, COST provides strong pillar for the brighter future in personalized medicine and healthy life. Policy makers should support these trends fast and strongly so that we could jointly define and introduce the really healthy life to all.

## References

[1] Louca S, Pochet R, Schinzer D (2012). Why another conference on personalized medicine?. Croat Med J.

[2] Louca S (2012). Personalized medicine – a tailored health care system: challenges and opportunities.. Croat Med J.

[3] Evers AW Rovers MM Kremer JA Veltman JA Schalken JA Bloem BR An integrated framework of personalized medicine: from individual genomes to participatory health care. Croat Med J 2012 53 301 3doi:10.3325/cmj.2012.53.301 22911520 10.3325/cmj.2012.53.301 PMC3428816

[4] Henney AM (2012). The promise and challenge of personalized medicine: aging populations, complex diseases, and unmet medical need.. Croat Med J.

